# Role of Cardiac Biomarkers in Cancer Patients

**DOI:** 10.3390/cancers13215426

**Published:** 2021-10-29

**Authors:** Gennaro Carmine Semeraro, Carlo Maria Cipolla, Daniela Maria Cardinale

**Affiliations:** 1 Cardioncology Unit, European Institute of Oncology, IRCCS^†^, 20145 Milan, Italy; daniela.cardinale@ieo.it; 2 Cardioncology and Second Opinion Division, European Institute of Oncology, IRCCS^†^, 20145 Milan, Italy; carlo.cipolla@ieo.it

**Keywords:** androgen deprivation, carcinoid, cardiac amyloidosis, cardiac biomarkers, cardio-oncology, cardiotoxicity, immune check-point inhibitors, NT-proBNP, predictive, troponin

## Abstract

**Simple Summary:**

Cardiac biomarkers have proved increasingly useful in the various branches of cardiology, not sparing the field of cardio-oncology. With specific reference to the latter subject, they have been investigated as predictors and/or diagnostic and monitoring tools, as well as prognostic factors, with the purpose of allowing the early prevention of many cardiovascular complications related to the direct action of some cancer types or related to the toxicity of its treatments. However, despite this great potential and excellent cost-effectiveness, their usefulness in some areas still seems to be limited due to lack of sufficient specificity or sensitivity. In fact, in clinical practice, while their use is nowadays standard in some circumstances, evidence does not yet support their routine use in other cases.

**Abstract:**

In patients with cancer—and especially some specific subtypes—the heart can be pathologically affected due to the direct action of the tumor or its secretion products or due to the toxicity of some oncological treatments. Cardiac biomarkers have been investigated as inexpensive and easily accessible tools for prediction, early diagnosis, monitoring, or prognosis of various forms of cancer-related cardiac diseases. However, their clinical usefulness was not always clearly demonstrated in every area of cardioncology. For the identification of anthracycline related cardiotoxicity in the very early stages troponins proved to be more efficient detectors than imaging methods. Nevertheless, the lack of a standardized dosage methodology and of cardiotoxicity specific thresholds, do not yet allow to outline the precise way to employ them in clinical routine and to incorporate them into appropriate diagnostic or managing algorithms. Cardiac biomarkers proved also effective in patients with primary cardiac amyloidosis, in which both troponins and natriuretic peptides were able to predict adverse outcome, and carcinoid heart disease, where a precise diagnostic cut-off for N-terminal prohormone of brain natriuretic peptide (NT-proBNP) was identified to screen patients with valvular involvement. Likewise, NT-proBNP proved to be an excellent predictor of postoperative atrial fibrillation (POAF). On the contrary, evidence is still not sufficient to promote the routine use of cardiac biomarkers to early diagnose myocarditis due to immune check points inhibitors (ICIs), radiotherapy induced cardiotoxicity and cardiac complications related to androgenetic deprivation. In this review we present all the evidence gathered so far regarding the usefulness and limitations of these relatively inexpensive diagnostic tools in the field of cardio-oncology.

## 1. Introduction

Heart disease secondary to cancer can be divided in two main strands on the basis of the causative mechanism: iatrogenic cardiac damage due to cancer treatments toxicity and direct cardiac damage caused by the tumor itself. In both conditions, cardiac biomarkers have been investigated for their potential predictive, diagnostic, monitoring and a prognostic role.

The prediction of left ventricular dysfunction (LVD) caused by chemotherapy with high anthracycline doses and the diagnosis of cardiotoxicity in an early subclinical stage, i.e., when it is still undetectable with standard cardiovascular imaging methods, are undoubtedly the clinical purposes for which the employment of cardiac biomarkers has been most analysed in cardioncology to date. However, even in this setting, a universal agreement on how to precisely make use of them into routine clinical practice has not been reached yet [1].

Similar difficulties in establishing the exact potential diagnostic and predictive role of cardiac biomarkers also emerged in relation to cardiotoxicity due to immune checkpoint inhibitors (ICIs) and radiotherapy [2,3].

On the other hand, the diagnostic role and usefulness of NT-proBNP as a screening tool for heart valves involvement in the context of carcinoid syndrome and as a predictor for lung cancer surgery related atrial fibrillation seem to be well established [4,5,6], as well as the prognostic role and the usefulness in monitoring cardiac amyloidosis course of the previously mentioned biomarker together with highly sensitive troponin T (hs-TnT) [7].

The purpose of this review is to present the evidence gathered so far regarding the usefulness and limitations of cardiac biomarkers in the various cardioncology fields, including those recently emerged, and also to provide insights for research where there are still gaps in the evidence.

## 2. Cardiac Biomarkers Phatophysiology

**Figure 1:**
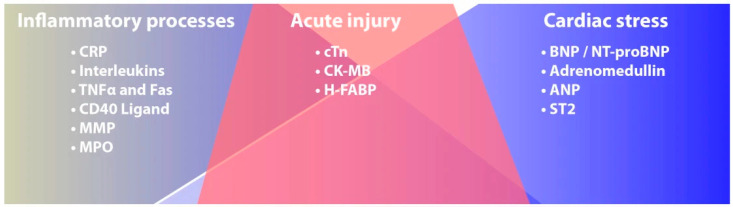
Main cardiac biomarkers and pathophysiological mechanisms involved in their plasma increase. Modified from McLean et al. [8].

The most used cardiac biomarkers in clinical practice are troponins (cTns) and natriuretic peptides (NP). Depending on the pathophysiological mechanism involved in each specific pathology, one or the other type of biomarker may be more useful depending on whether the cardiac involvement relies more on direct cellular damage or, conversely, on haemodynamic stress. (Figure 1) [8].

Troponins I (cTnI) and T (cTnT), are part of the contractile myofibrils of myocardial cells, and monitoring their release into blood circulation is an effective method to evaluate myocardiocytes integrity. While there is no evidence that cTnI could be released by non-cardiac tissues, in patients with myopathies damaged skeletal muscle releases proteins that can be detected by the cTnT assays, resulting in the latter being a slightly less specific marker of cardiac damage. [9] Since a variable low concentration of cTns can be detected even in the circulation of normal subjects, the diagnosis of myocardial injury relies on measuring a cTn value greater than the 99th percentile of the upper reference limit (URL) [10]. It can be acute, if characterized by a rapid increase and subsequent decrease in cTn values, or chronic, when cTn levels are constantly elevated. However, elevated cTn values do not indicate the precise mechanisms underlying myocardial damage and can be found not only during acute ischemic events, but also after various other inflammatory or mechanical insults or even in the absence of proved pathology (Table 1) [11].

**Figure 2:**
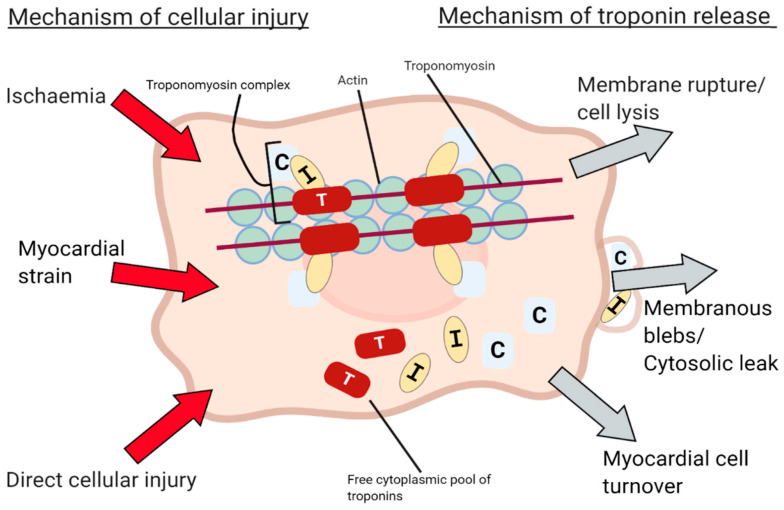
Mechanisms of Troponin release. From Taggart et al. [14].

Myocardial injury can be a manifestation of irreversible or reversible damage; in the first case the release of structural proteins from the myocardium relies mainly on apoptosis or necrosis of the myocardial cells; in the latter case, on the contrary, troponins release may occur in the absence of cell death and could be due to increased cell wall permeability or formation and release of membranous blebs. Other mechanisms of troponin release, in the absence of a myocardial damage, are normal myocardial cell turnover and cell release of cTn breakdown products (Figure 2). However, it is not clinically possible to distinguish the mechanisms that cause the cTn level to rise from the laboratory result alone [11,12,13,14].

Natriuretic peptides (NP)—atrial (ANP), cerebral (BNP) and NP type C (CNP)—are released into the circulation in response to haemodynamic stress or adrenergic activation. ANP is produced by the atria, BNP mainly by the ventricles. Their effect is mediated by various cell types and is achieved through the induction of vasodilation, natriuresis and diuresis. CNP is mainly produced by endothelial cells and its role in cardiovascular pathophysiology is less clear. All NPs are synthesized as pre-prohormones, which are cleaved into prohormones and then into biologically active hormones; residual inactive fragments can also be detected in plasma. As already mentioned, NPs are markers of mechanical stress. In fact, volume or pressure overload induces their synthesis; once secreted, they exert diuretic, natriuretic and vasodilating effects to mantain cardio-renal and hemodynamic homeostasis. This results in an improvement in myocardial relaxation and a reduction in vasoconstriction, Na + reabsorption and sympathetic hypertone that occur in case of heart failure (HF). Among the NPs, the active (BNP) and inactive (proBNP and NTpro-BNP) forms of BNP are classically used as biomarkers for the diagnosis and monitoring of acute and chronic heart failure due to their longer half-life and stability. It is demonstrated that BNP and NTproBNP levels directly correlate to clinical outcomes in patients with HF [15,16].

Sometimes the two types of markers can also increase simultaneously providing complementary or additive information. It was proved that the combined increase of both cardio-specific biomarkers increases cardiovascular risk several times even in asymptomatic individuals enrolled in the general population [17].

## 3. Iatrogenic Cardiac Damage Caused by Cancer Treatments

### 3.1. Cardiotoxicity

Cardiotoxicity is one of the major side effects of cancer treatments, affecting the overall prognosis of the patient and potentially causing chemotherapy discontinuation. In the past, its diagnosis relied at first on signs and symptoms of heart failure, and then on the detection of systolic left ventricle dysfunction (LVD) by cardiovascular imaging methods. However, this approach only allows for late diagnosis, when macroscopically evident organ damage has already occurred. Furthermore, the diagnostic accuracy of echocardiography for LVD detection, especially when it is mild, is compromised by operator-dependency, while more advanced imaging modalities with a greater reproducibility are limited by excessive cost and poor accessibility [18]. Since evidence from the last twenty years have demonstrated the possibility of preventing chemotherapy-related decrease in left ventricular ejection fraction (EF) by promptly initiating an adequate cardio-protection, it emerged the need for an earlier detection of cardiotoxicity with more refined diagnostic methods, or, alternatively, to identify predictors that could help select patients deserving cardioprotective treatments [19,20,21,22]. Therefore, considering that they are inexpensive, easily detectable and reproducible, and that they reflect even minimal cardiomyocytes damage or slight haemodynamic fluctuations, circulating cardiac biomarkers have been investigated as potential early cardiotoxicity indicators and/or predictors of subsequent LVD onset [18].

While natriuretic peptides have a high intra-individual variability of about 50–60%, intra-individual variability between two consecutive measurements of hs-cTnI or hs-cTnT is low (about 9%); given this, a difference in plasma concentration between two distinct dosages of the same troponin subtype in a single patient can be considered statistically significant when is greater than 30%, whatever the assay used [17,23]. This is relevant given that in clinical practice there is wide variability in cut-off values between the several assays of hs-cTnI and hs-cTnT [23,24,25]. It should be emphasized that, in light of what has been said, an increase between two measurements just greater than 3–5 ng/L in a time frame of a few weeks could be statistically significant. This increase of 3–5 ng/L in hs-Tn concentration is correlated to a necrosis of about 10–20 mg of myocardial tissue, absolutely undetectable with cardiac imaging techniques, even with the most sensitive ones [17,23,26]. This important experimental evidence suggests that when using hs-Tn for cardiotoxicity detection, biomarker measurement using a hs-cTnI or hs-cTnT method should be performed in all patients with a blood sample collected at baseline, prior to treatment with cardiotoxic agents, and that monitoring of biomarker changes during the whole period of treatment must be performed using the same hs-cTnI or hs-cTnT method (possibly in the same clinical laboratory) [17].

#### 3.1.1. Anthracycline Cardiotoxicity

**Figure 3:**
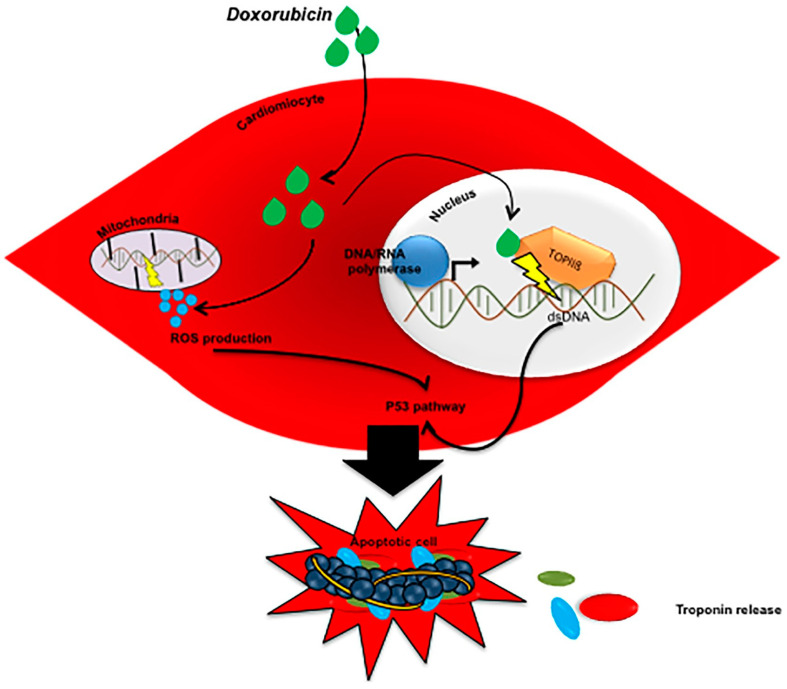
Mechanism of troponin release caused by anthracyclines [27].

Anthracyclines exert their cytotoxic effect by binding to isoenzymes of topoisomerase 2 which are inhibited in cardiomyocytes with consequent double-stranded DNA breaks. This results in activation of the p53-mediated apoptotic cell death pathway. Anthracyclines also cause an increased release of reactive oxygen species (ROS), which act as secondary signaling molecules in various pathways involved in homeostasis, including cell proliferation and cell death. In the heart, both of the above mechanisms participate in determining cell lysis with the release of troponin (Figure 3) [27].

Troponin as a cardiotoxicity predictor began to be investigated between the late 1990s and the 2000s, both in animal models and in patients undergoing chemotherapy with high-dose anthracycline-containing regimens [28,29,30,31,32].

**Figure 4:**
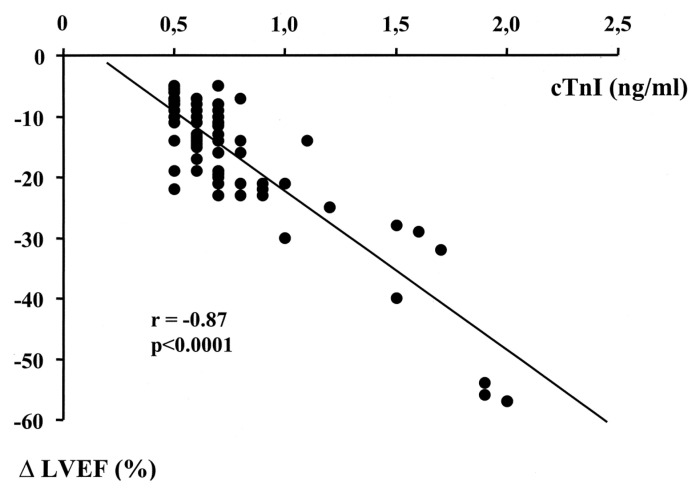
Linear correlation between TnI peak detected during chemotherapy and subsequent % EF drop [33].

Cardinale et al. showed that Troponin I (TnI) serially assayed at every cycle exceeded at least once the cut-off in 32% of the 204 patients enrolled in their clinical trial. Then, depending on troponin positivity, patients were divided into TnI positive and TnI negative; both groups experienced an early, mild decline in EF at the end of chemotherapy, but this decline turned out to be reversible only in patients without troponin elevation. A strong linear correlation was also found between the maximum troponin value detected during chemotherapy and the EF nadir reached during follow-up (Figure 4) [33].

Although this result was confirmed by some subsequent clinical trials, other authors did not find any significant increase in troponin related to anthracycline treatment. It is worth noting, however, that in many studies the biomarker was not dosed with every chemotherapy cycle, but often only after the end of the treatment, suggesting that its increase occurs acutely for the cardiac injury caused by cancer treatment and that the possibility of finding positive samples relies on the correct timing of the measurement [28,34,35]. Furthermore, Feola et al., in their study including 53 patients with breast cancer, while actually observing a significant mean troponin I rise from baseline (*p* = 0.0001) at the end of chemotherapy, failed to demonstrate that troponin increase had a good positive predictive value for the future development of LVD [36].

The use of troponin as a diagnostic marker and predictor of cardiotoxicity has not been questioned only because of the previously mentioned conflicting results. The lack of multicenter studies and the use of reference values approved for myocardial ischemia and not specific for the disease in question were also the subject of discordance [37]. Besides, further uncertainties arose after the introduction of ultra-sensitive troponin, but they were partially cleared thanks to a study by Salvatici et al. who found a good correlation and agreement between the old and the new assay [38].

To our knowledge, only two meta-analyses aimed at verifying the usefulness of troponin as a predictor of cardiotoxicity were carried out, one including adult patients, one including pediatric patients. It emerged that in both cases anthracycline chemotherapy actually can induce a release of troponin with a consensual plasma level increase. Adult patients who were treated with beta-blockers and angiotensin converting enzyme inhibitors during chemotherapy had a significantly lower mean serum troponin peak than untreated patients (OR 4.1). Moreover, in the adult population, patients with positive troponin assays were more at risk of developing left ventricular dysfunction and troponin was shown to have a high negative predictive value (93%) for a future EF decrease; on the contrary this evidence was not strong enough for pediatric patients [39,40].

Finally, a study by Kang et al. analysed the predictive capacity towards cardiotoxicity of the combination of a biohumoral datum—hs-cTnI rise—and a cardiovascular imaging datum—decrease in GLS—demonstrating a good positive (61%) and excellent negative (95%) predictive value [41].

It must be said that regardless of the predictive capacity of troponin for clinically manifest cardiotoxicity, its increase alone represents an index of subclinical cardiac damage related to cancer treatment. In light of this, and given that some clinical trials such as OVERCOME and PRADA [19,20] demonstrated the efficacy of the intrachemotherapeutic administration of drugs such as angiotensin converting enzyme inhibitors (ACE-I) and beta-blockers in preventing the development of LVD due to cardiotoxicity, some authors proved that a strategy based on prompt administration of cardioprotective therapies exclusively in patients having an increase in troponin during chemotherapy was effective as well [21,22].

Other markers that have been extensively studied as possible predictors or early detectors of cardiotoxicity are natriuretic peptides. For them, however, the results of the studies were not as encouraging as for troponin. Little evidence of their usefulness in this context comes from a study by Feola et al., which showed that the mean baseline BNP of patients who experienced a decrease in EF of more than 10 percentage points from baseline after chemotherapy was significantly higher than in patients whose EF did not change [36]. However, no other relevant evidence has emerged supporting their effectiveness as subclinical cardiotoxicity diagnostic tools. In the aforementioned meta-analysis by Michel et al. no consistent association between an increase in BNP or N-terminal-proBNP (NT-proBNP) during chemotherapy and subsequent LVD development was found [39].

**Figure 5:**
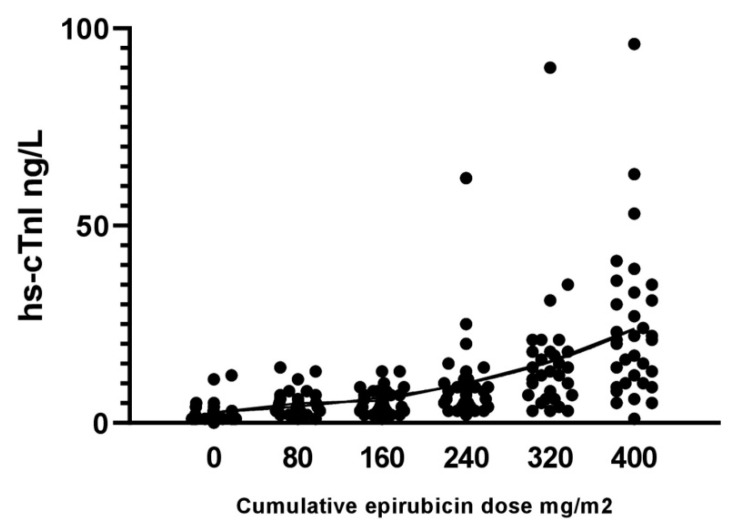
Troponin progressive increase as cumulative anthracycline doses increases with chemotherapy advancement. From Tzolos et al. [42].

The different effectiveness of troponins and natriuretic peptides as early markers of cardiotoxicity, as easily understandable, lies in the different mechanisms by which they are released into the circulation. The increase in the blood concentration of natriuretic peptides mainly depends on the haemodynamic stress to which the atria are subjected; this stress usually occurs when ventricular function is already significantly impaired. Troponins, on the other hand, are released into the circulation early in the event of cellular distress, even in the absence of cell death. The gradual progressive increase in serum troponin from baseline observed in the various clinical trials as chemotherapy progresses demonstrates its unequivocal dependence on the cumulative toxic effect caused by each subsequent anthracycline administration (Figure 5). The lack of a specific cut-off for cardiotoxicity, the early use of preventive cardioprotective drugs in some studies and some additional individual patient factors could explain the reason for the low positive predictive value of troponin despite the high negative predictive value for manifest LVD found in meta-analyses [18,42].

#### 3.1.2. Trastuzumab Cardiotoxcity

**Figure 6:**
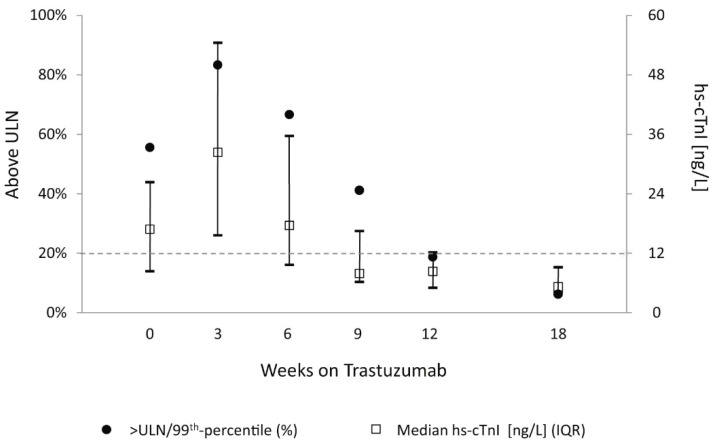
Troponin behavior during trastuzumab therapy following previous chemotherapy with other drugs including anthracyclines [45].

In 2010 Cardinale et al. carried out a study including 251 women with breast cancer that investigated troponin as a predictor of future LVD related to trastuzumab treatment. Troponin was found to be a predictor of LVD with a hazard ratio (HR) of 22.9 (*p* < 0.001). Furthermore, patients with troponin increase had less chance of recovery from LVD than those who experienced a drop in EF but no troponin level rise. It should be underlined that some of the patients had previously been treated with other chemotherapeutic agents, including anthracyclines, and trastuzumab was often administered in association with other anti-cancer drugs. The same authors described a troponin increase at baseline in 7 of the 36 patients who overall tested positive for troponin during treatment with trastuzumab. In addition, in most cases, troponin increased during the very first courses of trastuzumab, and then decreased during treatment continuation. This would suggest that troponin increase was due to delayed myocardial damage from previous chemotherapy with other cardiotoxic drugs, rather than direct damage from trastuzumab [43,44]. A similar troponin behavior during trastuzumab courses was found by Dhesy-Thind et al. in a recent clinical trial where 21 women with HER-2 positive early-stage breast cancer were enrolled after completion of an anthracycline-based chemotherapy. The hs-cTnI concentrations in the cohort were the highest at 3 weeks after the initiation of Trastuzumab but then, started to decrease, reaching the lowest at 18 weeks while still on therapy (*p* < 0.01) (Figure 6) [45].

Zardavas et al. also found a correlation between high troponin levels at baseline and subsequent LVD development in patient treated with trastuzumab; similarly to what was observed in the study by Cardinale et al., only a really small fraction of patients suffered from an increase in troponin during trastuzumab courses [46].

Ponde et al. investigated troponin and NPs ability to predict LVD in anthracycline naïve patients treated with trastuzumab and found that their increment is very rare during such therapy [47].

Overall, evidence suggests that troponin is effective in predicting cardiotoxicity in patients undergoing sequential treatment with anthracyclines followed by trastuzumab, where the latter appears to act as a precipitating agent. However, troponin, and cardiac biomarkers in general, do not appear to be clearly useful for cardiotoxicity monitoring during treatment with trastuzumab not combined with anthracyclines. The reason why troponin does not significantly increase when on treatment with trastuzumab alone may lie in the mechanism by which this drug exerts its cardiotoxic action, which does not imply cell death. As for natriuretic peptides, their limited usefulness could be related to the fact that significant increases in their levels during trastuzumab courses occur only if the patient develops severe LVD, but usually milder dysfunctions are detected earlier with imaging techniques.

#### 3.1.3. Myocarditis Due to Immune Check Points Inhibitors

Immune checkpoint inhibitors (ICIs) are anticancer drugs that disinhibit T-cells by interfering with checkpoint molecules, leading to an enhanced antitumor immune response. They can cause autoimmunity also against the myocardium.

A study by Mahmood et al. on 964 patients treated with ICIs it showed that the incidence of myocarditis was 1.14%, while that of major adverse cardiovascular events (MACEs) was 0.52% [48].

Mir et al. carried out in 2018 a systematic review on cardiac complications associated with ICIs use. The authors were able to gather data relating to the onset of heart disease in patients treated with these drugs only from case reports and small case series, since none of the randomized clinical trials under analysis reported data on cardiac complications. Therefore, no conclusions could be drawn on the overall incidence of these complications. The authors then focused on 99 patients who developed signs of cardiotoxicity, without specifying in detail any definition of the latter and probably referring to any form of acute cardiac involvement. In fact it is then specified that among these 99 patients 45% were diagnosed with myocarditis, while 27% were affected by other forms of acute heart failure or cardiomyopathy with no signs of myocarditis; moreover, 15% had pericarditis and 12% conduction disturbances. The mortality rate among this 99 patients was 35%, which indicates the relevance of ICIs cardiotoxicity and the importance of an early diagnosis despite the overall low incidence found in other studies. When Troponin T was dosed, it was elevated in 93% of cases. Creatine kinase (CK) and its MB isoform (CK-MB), BNP and myoglobin levels were found to be above the threshold in all cases [49,50].

Since ICIs-related myocarditis can be subtle in onset but potentially lethal, the importance of early diagnosis even in the subclinical phase—in the absence of clear clinical signs and symptoms—is fundamental to promptly intervene. Cardiac biomarkers were investigated as potentially optimal diagnostic tools for this purpose. Research focused on the ability of the same markers to predict, if dosed at baseline, or early detect, if dosed serially during chemotherapy, myocarditis at the onset. Among the few clinical trials regarding the predictive ability of baseline troponin measurement for subsequent myocarditis after ICIs initiation, none proved a good positive predictive value or good specificity of the biomarker [3].

**Figure 7:**
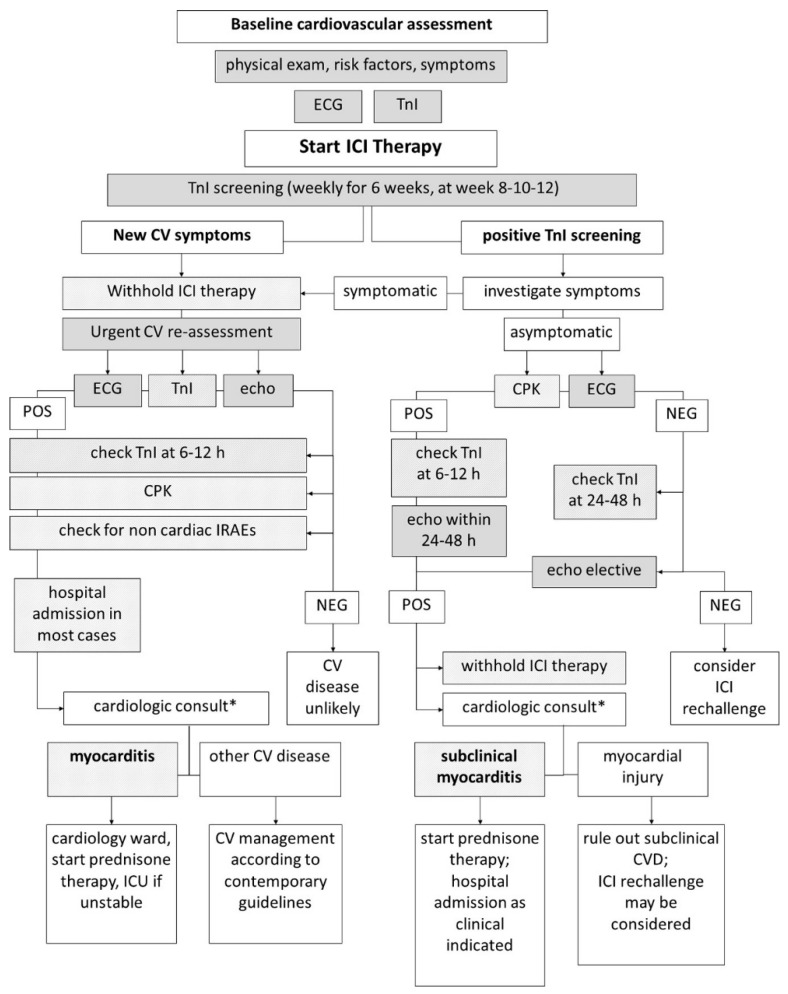
Algorithm for early detection of ICIs related myocarditis proposed by Spallarossa et al. The timing of troponin assays repetition differs due to the presence/absence of symptoms. * Result of integration and interpretation of clinical, anamnestic, laboratory, ECG and echocardiographic data [53].

The serial measurement of troponin during ICIs courses has also been proposed in routine clinical practice [51], but the clinical usefulness of a biomarker-based screening for ICIs-related myocarditis has not yet been proved [3]. To be useful, this approach should lead to the early diagnosis of myocarditis without resulting in the unnecessary suspension of cancer therapy. Since myocarditis is not the only complication of ICIs therapy potentially causing troponin increase, Spallarossa et al. suggested to improve the specificity of a biomarkers-based screening strategy for myocarditis by adding the measurement of CK besides troponin. In fact, a generic myocardial lesion not related to myocarditis has less probability to cause an increase of less sensitive markers of myocardial damage like CK itself; however, CK is not a specific cardiac marker, since it increases also in case of myositis, a possible side effect of ICIs. Spallarossa et al. also proposed an algorithm for ICIs-related myocarditis screening based on cardiac biomarkers (Figure 7) [52,53].

However, in their first study Spallarossa et al. have not identified a precise troponin cut-off specific for myocarditis, while they adopted the standard threshold used to detect myocardial injury. The study included 59 patients treated with nivolumab for non-small cell lung cancer. Six patients had a troponin value above the threshold but only one was deemed as possibly having subclinical myocarditis [52,54]. Given the high risk of inappropriate therapy interruption in the other five patients with high troponin levels but without evidence of a threatening cardiac complication, the same authors concluded that a screening strategy based on troponin needs to be better investigated through larger clinical trials [52].

Waliany et al. in a 2021 carried out a prospective study and found that among 214 patients included, 11.2% had a positive hsTnI value (≥55 ng/L) during chemotherapy with ICIs, while the incidence of myocarditis was only 1.4% (3 patients). In most cases, therefore, hsTnI positivity was then attributed to cardiovascular causes other than myocarditis. Using different hsTnI thresholds the positive predictive value (PPV) for myocarditis was 12.5% for 55 ng/L, 75.0% for 1000 ng/L, and 100% for 2000 ng/L; the negative predictive value (NPV) was 100% at each threshold. It must be specified that the diagnosis of myocarditis was only “probable” in one case and “possible” in another. Moreover, no patient showed a reduction in EF [55]. This is the largest prospective study carried out so far on the topic. Other few studies were carried out, but they were weak in methodology and had a too small population to find significant results [3].

Unlike what happens for anthracycline cardiotoxicity, where the eventual increase in troponin is slow and progressive with the advancement of the chemotherapy cycles (a sort of long-lasting troponin curve that slowly returns to baseline after the end of the therapy), a troponin elevation due to myocarditis related to ICIs is expected to be sudden, reflecting the acuteness of the triggering event. By consequence it is difficult to establish the correct timing of the dosage to promptly detect such an acute increase in troponin. Moreover, a sudden hyper-troponinemia in the absence of symptoms or clear clinical and instrumental signs does not allow by itself a distinction among the underlying mechanisms, i.e., myocarditis or other causes (arrhythmias, pulmonary embolisms, myocardial infarctions, etc.) or even no specific evident cause. Although Waliany et al. were able to identify a troponin cut-off with a high positive and negative predictive value for ICIs related myocarditis, given the overall low incidence of this complication, such a result cannot be considered strong evidence, as it was obtained from a small population sample (only three patients were diagnosed with myocarditis) and it is not yet possible to draw definite conclusions on the net clinical benefit that a troponin-based monitoring approach would entail in everyday clinical practice. Although promising, therefore, specific troponin cut-offs for ICIs related myocarditis need to be validated by larger clinical trials and, in general, the effectiveness of a screening method based on troponin in terms of obtaining aa prompt diagnosis with low risk of inappropriate therapy interruption needs to be confirmed as the same authors stated in the conclusions [55].

Some clinical trials showed also that elevated NT-proBNP is a very frequent finding during ICI-associated acute myocarditis and that elevated levels of natriuretic peptides can be found in virtually 100% of patients with ICI-related cardiotoxicity. However, the specificity of natriuretic peptides remains as low as for troponin [56].

To date no precise protocol has been universally accepted for active screening of ICI-associated myocarditis [3]. According to the guidelines of the American Society of Clinical Oncology, troponin should be dosed upon symptoms, while it could be measured at baseline especially in patient treated with combination immune therapies; however, it is not clearly specified whether measurements should be performed serially during chemotherapy courses and at what timeframe [57].

### 3.2. Radiotherapy

Most of the studies aimed at evaluating the effects of radiotherapy on the heart using biomarkers have focused on the acute phase, with the aim of detecting immediate subclinical damage expressed precisely as fluctuations in the biomarkers themselves. On the other hand, since the anatomical and functional alterations that radiotherapy can induce are usually extremely late, it is more difficult to demonstrate a correlation between the subclinical damage detected by the biomarker alterations and the future development of myocardial or valvular pathology

In 1995 Hughes-Davies et al. investigated whether troponin T values changed from baseline immediately after left breast irradiation; they found no signs of acute myocardial injury after radiotherapy, at least within the detection capability of the assays available at the time [58].

In 2010 Nellessen et al., after dosing serially during the various cycles of radiotherapy treatment TnI and BNP, showed that both biomarkers had increased significantly when plotted on a log scale (log_10_), even if changes were not so relevant when absolute values were considered [59].

Zaher et al. investigated whether there were differences in the extent of heart damage related to the side on which radiotherapy for breast cancer was performed. A significant change in mean EF occurred only in patients treated on the left side. Six of these patients had a one-year drop in EF of 20% or more. TnI and creatine kinase MBRI isoenzyme values were more frequently above threshold in left breast treated patients, and the six patients with an EF drop ≥ 20% were all among those with altered biomarkers and treated on the left side [60].

**Figure 8:**
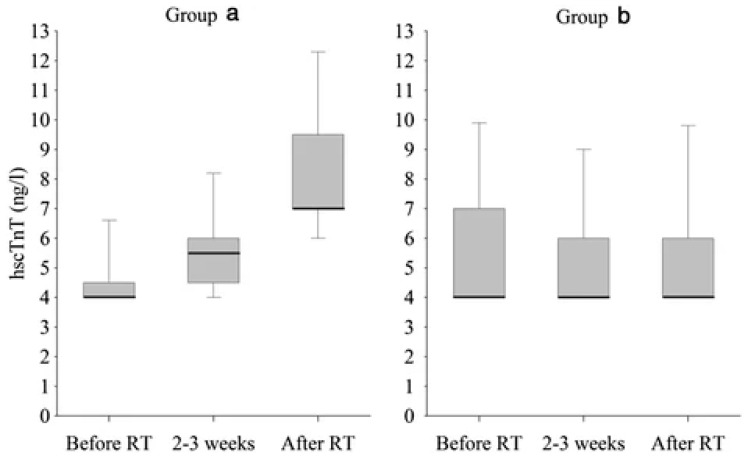
Troponin level variation before, during and after RT in patients treated with higher radiation doses and who had a hscTnT increase > 30% from baseline (Group a) versus patients treated with lower radiation doses and who did not experience a significant increase in hscTnT (Group b) [61].

In 2015 Skyttä et al. carried out a prospective study involving 58 patients with left breast cancer treated with radiotherapy and naive to chemotherapy. The authors measured TnT before, during and at the end of radiotherapy and considered an increase in hscTnT > 30% to be significant. This increase from baseline occurred in 21% of patients during or after radiotherapy. The group of patients with increased hscTnT received significantly higher radiation doses than patients with no increase in hscTnT (Figure 8). BNP did not increase in a significant way after radiotherapy and there was no difference between the two groups [61].

In a subsequent prospective study, the same authors evaluated radiotherapy-induced changes in cardiac biomarkers and echocardiographic parameters from baseline over a three-year period. At the end of the follow-up, mean global longitudinal strain was significantly reduced, as well as EF and cardiac output. The diastolic function of the left ventricle was also adversely affected, as well as the size of the left atrium. These changes were more prominent in patients treated for left breast cancer than right breast cancer. On average, NT-proBNP values increased significantly three years after radiotherapy. However, the same marker did not increase in patients treated on the right side [62].

Some other studies did not show significant changes in biomarkers levels after radiotherapy. Donovan et al. measured highly sensitive TnI and TnT before and during radiotherapy at week two and four; they found no significant increase in the two markers, but the study population was very small and the cumulative radiation dose was low [63]. De Sanctis et al. found no increase in cardiac TnI and BNP levels from baseline related to left breast irradiation [64]. Yu et al. investigated whether radiotherapy could induce high sensitivity TnI increases after anthracyclines and trastuzumab based chemotherapy; no significant change in biomarker level was detected from baseline [65]. Furthermore, Saibene et al. investigated if intraoperative radiotherapy can cause heart damage by dosing ultrasensitive TnI and NT-proBNP levels at baseline, six hours after the end of surgery and after 12 months. None of the patients showed altered levels of either biomarker before or after surgery or during follow-up [66]. This result has been recently confirmed by Stefanovic et al. [67].

Overall, the results of the studies on cardiac biomarkers behaviour during or immediately after radiotherapy are conflicting, although apparently there is some degree of evidence that, for high cumulative radiation doses in patients treated for left breast cancer, an increase in TnI can potentially occur as a sign of acute myocardial injury. As far as we know, there is no meta-analysis on this subject that could resolve the conflict in evidence [2]. Furthermore, there is too little evidence regarding the eventual correlation between an increase in cardiac biomarkers after radiotherapy and echocardiographic changes or cardiovascular events in the short-term. Finally, if any subclinical cardiac injury recorded in the acute phase could correlate with long-term cardiovascular morbidity or mortality is not clearly demonstrated and will require longer follow-up studies. [2,62,68].

### 3.3. Postoperative Atrial Fibrillation/MACEs

Another widely investigated application of cardiac biomarkers in oncology is that aimed at the prediction of postoperative atrial fibrillation (POAF) and other cardovascular events in patients treated with surgery for cancer, and particularly for lung cancer. Several studies were carried out for both types of biomarkers, natriuretic peptides and troponins.

Cardinale et al. in 2007, after dosing NT-proBNP 24 h before and 1 h after lung cancer surgery, found that the incidence of POAF was markedly increased in those patients with a high level of this marker in any of the two measurements (relative risk respectively was 27.9 and 20.1) [69]. In a study by Nojiri et al., the preoperative BNP value was found to be an effective predictor of POAF in patients undergoing lung cancer surgery; with a cut-off of 30 mg/mL, this marker showed a sensitivity of 77%, a specificity of 93%, a positive predictive value of 81% and a negative predictive value of 92%. [70]. 

Although some other studies did not show consistent results with the previously cited trials [71,72], two meta-analyses proved that natriuretic peptides are good predictors of POAF: according to the results obtained by Cai et al., summary estimates for the sensitivity and specificity of using NP levels for predicting POAF were 75% and 80% respectively, while from Simmers et al. meta-analysis including only patients scheduled fot thoracic surgery it merged that an elevated preoperative NP measurement was associated with an OR of 3.13 for POAF [4,5].

Regarding the ability of troponin to predict POAF, as far as we know, all the studies carried out so far have included only patients undergoing cardiac surgery. Furthermore, the results of the various clinical trials are not in agreement and there is a lack of meta-analyses. In two studies, troponin did not predict POAF [71,73]. On the contrary, Leale et al. found that it was significantly more frequent to find TNI, dosed immediately after coronary artery bypass grafting, beyond the cut-off of 0.901 ng/mL in patients who subsequently developed POAF than in those who remained in sinus rhythm. [74]. Lahoz-Tornos et al. demonstrated that high troponin T values detected before surgery could predict POAF but with modest sensitivity and specificity [75]. Hernández-Romero et al. in 2013 demonstrated that high pre-surgical troponin T values were independent predictors of POAF, while post-surgical values were not, suggesting that intra and postoperative myocardial damage does not provoke this arrhythmia [58].

Despite the lack of data regarding the ability of troponin to predict POAF in patients undergoing thoracic surgery, a recent study has shown that in this population an increase in TNI over 0.16 ng/mL in the early postoperative period is however correlated with an increase in mortality within one year [76].

### 3.4. Androgenic Deprivation in Prostate Cancer Patients

Androgen deprivation therapy is associated with an increased risk of cardiovascular events. In particular, gonadotropin-releasing hormone (GnRH) agonists appear to be more prone to causing such complications than antagonists [77]. The impact of androgen deprivation on the heart represents, therefore, a new topic on which research is recently focusing to evaluate the usefulness of cardiac biomarkers as prognostic tools or predictors.

In 2016 Campora et al. found an association between higher levels of NT-proBNP and TnT dosed 3 months after initiation of abiraterone therapy and the incidence of severe cardiac adverse events [78].

Margel et al. investigated whether serum NT-proBNP and high-sensitivity troponin levels could predict the onset of new cardiovascular events during a 12-month period in prostate cancer patients with a history of previous cardiovascular disease who were treated with a GnRH agonist or antagonist. The baseline features between patients enrolled in the two treatment arms were similar. High NT-proBNP and troponin baseline levels were associated with the occurrence of new cardiovascular events in the GnRH agonist group but not in the antagonist. There were no significant changes in NT-proBNP and troponin from baseline either in patients who experienced a new cardiovascular event, or in those who did not, during the 12-month follow-up [77,79].

Another ongoing study aimed at evaluating the difference between GnRH agonists and antagonists in terms of cardiac side effects provoked, in addition to investigating the incidence of adverse cardiovascular events induced by the two different drug typologies, will analyze if cardiac biomarkers are useful in this context [80].

Despite the evidence gathered so far, further studies are needed to clearly establish if NT-proBNP can effectively predict cardiovascular complications in patients undergoing androgen deprivation.

## 4. Cardiac Involvement in Oncological Diseases

### 4.1. All-Cause Mortality in Newly Diagnosed and Chemotherapy-Naïve Cancer Patients without Evidence of Acute Cardiac Disease

**Figure 9:**
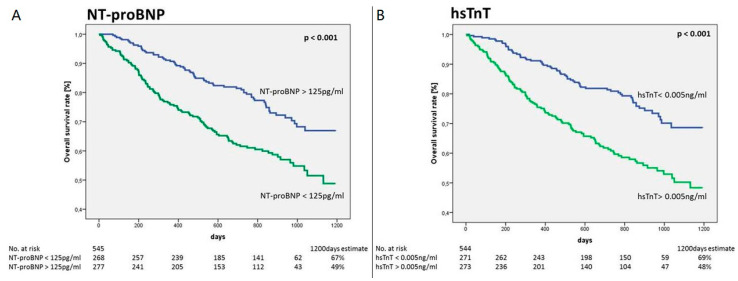
Prognostic value (Kaplan–Meier estimates) for all-cause mortality of (**A**) N-terminal pro B-type natriuretic peptide (NT-proBNP) with a threshold of 125 pg/mL and (**B**) hsTnT with a threshold of 0.005 ng/mL for patients with newly diagnosed tumour disease (*p* < 0.001 between two groups for NT-proBNP and hsTnT, log-rank test). Modified from Pavo et al. [82].

Elevated levels of natriuretic peptides, hsTnT and other cardiac disease-associated molecules can occasionally be found in the plasma of newly diagnosed and chemotherapy-naïve cancer patients without known previous heart pathology or affected also by stable cardiac pathologies but without clinical signs of acuities. Since the meaning of this isolated finding is unknown [81], Pavo et al. tried to test whether baseline increase in cardiac biomarkers in this population correlated with an increase in subsequent all-cause mortality. According to the authors, in fact, the detection of elevated levels of these cardiac markers could reflect the deleterious subclinical effects that cancer can have on heart, and could also correlate with the worsening of the oncological disease, assuming that cardiac involvement increases as the cancer progresses. In other words, cardiac biomarkers could represent not only indicators of heart involvement, but also, indirect prognostic tools for global outcome in cancer patients. More than 500 patients without cardiovascular disease or with a clinically stable cardiac condition and recently diagnosed with cancer of any type prior to the initiation of any cardiotoxic chemotherapy, were selected. Biomarkers were dosed at the first hospital presentation. During the follow-up, which lasted an average of 25 months, 34% of patients died. Among the various markers analyzed, also natriuretic peptides and hsTnT proved to be indipendent (regardless of age, sex, extent and stage of the tumor and the presence of comorbidities) predictors of mortality (Figure 9) [82].

Moreover, natriuretic peptides and hsTnT levels were found to be significantly higher (*p* = 0.002 and *p* < 0.001 respectively for NT-proBNP and hsTnT for the mean difference between stage 1 and stage 4) with progression of the tumor stage, suggesting that the presence of subclinical myocardial dysfunction and damage directly related to oncological disease progression [82].

Another very recent study including 930 patients with cancer (mainly breast cancer 48.4%, upper gastrointestinal carcinoma 10.6%, multiple myeloma 5.5%), with or without concomitant heart diseases and not yet undergone chemotherapy, failed to demonstrate baseline echocardiographic parameters or elevation of NT-proBNP correlate with all-cause mortality (logistic regression LVEF <50%: *p* = 0.46, NT-proBNP: *p* = 0.16), but proved that hs-cTnT above the median (≥7 ng/L) dosed prior to chemotherapy induction was an independent predictor of mortality (multivariant logistic regression, OR: 2.21, *p* = 0.0038). No death was clearly correlated to a direct cardiovascular cause [83].

Another study involving 5032 cancer-free patients found an association between baseline elevated levels of circulating biomarkers of inflammation, immune activation, metabolism, and fibrosis—all related to an altered cardiovascular homeostasis—and the increased incidence of subsequent cancer and cancer related-mortality, suggesting a shared pathway in the genesis of both cardiovascular and oncological pathologies and that biomarkers of subclinical cardiovascular damage may also indirectly have a predictive and prognostic value in relation to cancer. [84].

In conclusion, the employment of cardiac biomarkers as prognostic tools not only for cardiac outcomes in cancer patients but also for cancer-related mortality itself seems to be promising, but further evidence is needed to allow their standardized clinical use in this field.

### 4.2. Carcinoid Heart Disease

Cardiac biomarkers have been extensively investigated as diagnostic tools and predictors in carcinoid heart disease.

Based on the fact that serotonin typically secreted by functional neuroendocrine tumors is a potent vasoconstrictor, and therefore a potential ischemia-causing agent, Meijer et al. in 1999 investigated whether TnI, TnT and creatine kinase MB isoenzyme were able to detect myocardial damage in 20 patients with carcinoid syndrome. Patients were divided into 3 groups according to the degree of cardiac involvement established on the basis of echocardiographic parameters. All 20 patients showed increased urinary 5-HIAA excretion. TnI and TnT for all three groups were below detection limits. Creatine kinase values were also within reference limits [85].

**Figure 10:**
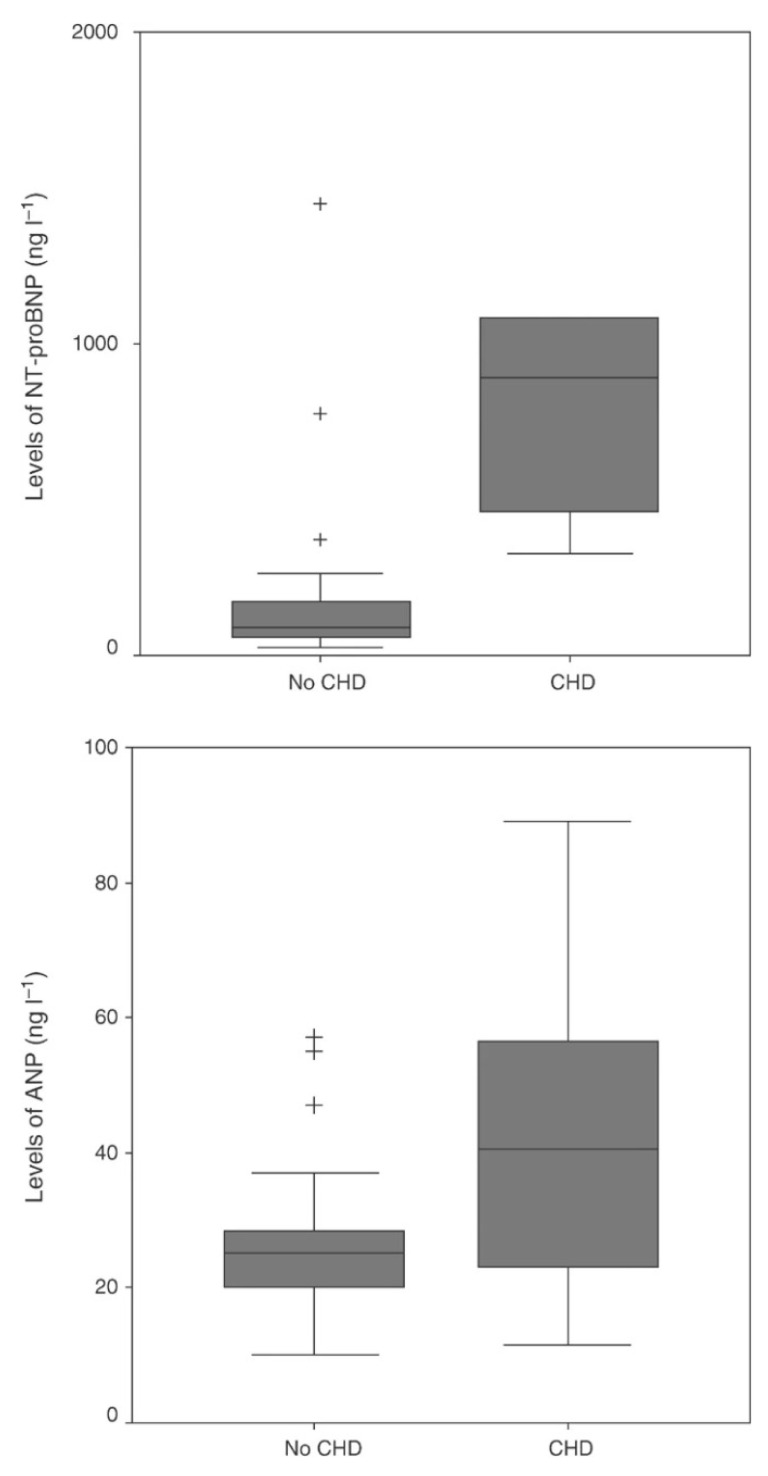
Difference in mean NT-proBNP (top box) and ANP (bottom box) levels between patients with carcinoid heart disease (CHD) and without (No CHD). Values beyond the lines are considered outliers (+) [86].

**Figure 11:**
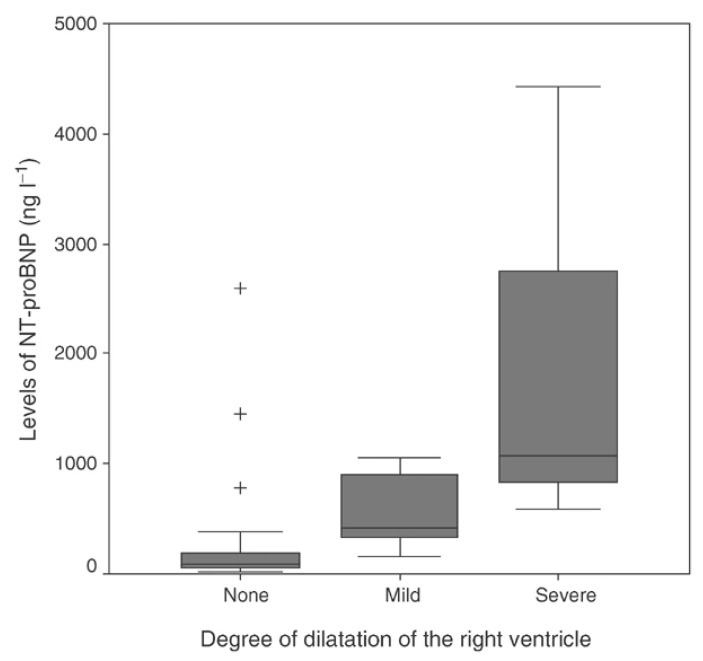
Correlation between severity of right ventricular dilatation and NT-proBNP values. Values beyond the lines are considered outliers (+) [86].

From then on, studies focused on natriuretic peptides. Zuetenhorst et al. in 2004 dosed Cromogranin A (CgA), 5-Hydroxyindoleacetic acid (5-HIAA), ANP and NT-proBNP in 32 patients with neuroendocrine tumors. The plasma levels of these markers were compared with echocardiographic data. Carcinoid heart disease was diagnosed in the presence of tricuspid valve thickening and a regurgitation of grade 3 or 4 out of 4 (28% of the patients satisfied these criteria, all with carcinoid related symptoms). Mean NT-proBNP and 5-HIAA levels were significantly higher in affected patients, while ANP and CgA levels did not differ between unaffected and affected patients (Figure 10). Right heart chambers dilation, tricuspid valve thickening, and regurgitation severity correlated significantly with NT-proBNP levels (Figure 11). Significantly better survival was observed in patients with normal NT-proBNP [86].

**Figure 12:**
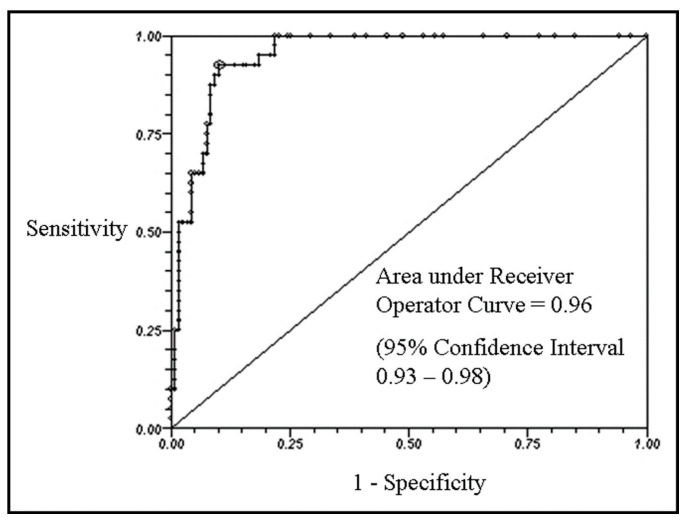
Receiver operator curve of NT–proBNP for diagnosis of carcinoid heart disease [6].

In 2008 Bhattacharyya et al. subjected 200 patients with carcinoid syndrome to echocardiography to rule out cardiac involvement. They also devised a score to quantify the severity of carcinoid heart disease. NT-proBNP, measured at the same time as echocardiography, was significantly higher in patients with cardiac involvement than in those without. The sensitivity and specificity of NT-proBNP for the detection of carcinoid heart disease with a cut-off of 260 pg/mL were 0.92 and 0.91, respectively, with an area under the receiver operating characteristic curve of 0.96. Furthermore, the levels of NT-proBNP correlated positively with the score designed by the authors (r = 0.81) (Figure 12) [6].

Similar results regarding the sensitivity and specificity of NT-prBNP with respect to carcinoid heart disease were found by other authors [87,88]. Korse et al. also demonstrated the prognostic potential of NT-proBNP in combination with CgA given the poorer survival of patients with both positive markers compared to patients with negative markers [87].

Based on what has been mentioned so far, it emerges that NT-proBNP is an excellent diasnostic and prognostic tool for carcinoid heart disease. To the best of our knowledge, no meta-analysis has been carried out on this subject, but the studies are all in agreement.

**Figure 13:**
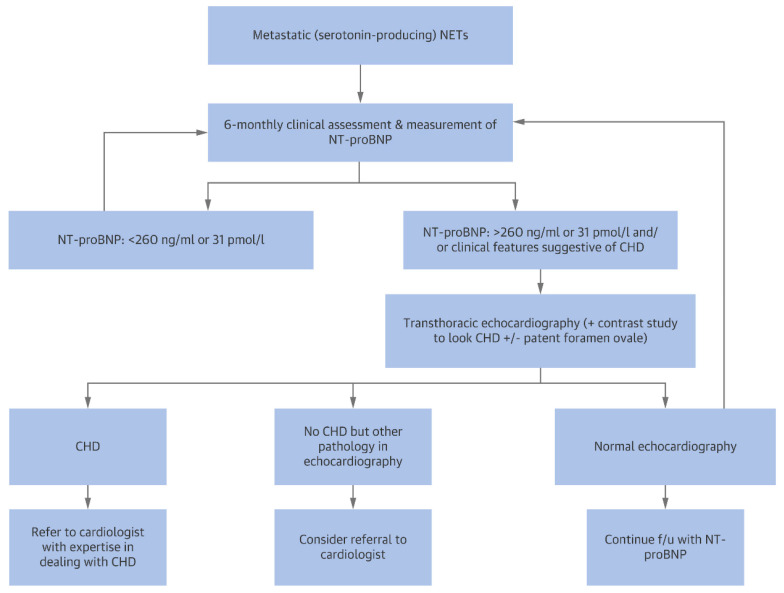
Screening algorithm for carcinoid heart disease (CHD) based on NT-proBNP values [89].

According to the “Expert Statement on Diagnosing and Managing Carcinoid Heart Disease in Patients With Neuroendocrine Tumors”, NT-proBNP is the most useful biomarker to screen for cardiac involvement in patients with carcinoid syndrome (Evidence Level 2 to 3, Grade B). The authors also proposed a screening algorithm for carcinoid heart disease based on the positivity of the same biomarker (Figure 13) [89].

### 4.3. Cardiac AL Amyloidosis

The first studies aimed at analyzing the levels of circulating cardiac biomarkers in patients with primary cardiac amyloidosis date back to the early 2000’s. Miller et al. demonstrated that troponins are above the ischemia threshold in most of the patients with cardiac involvement and no coronary disease [90]. In 2003 Dispenzieri et al. retrospectively investigated if troponins could predict a higher mortality in these patients and demonstrated that troponin T was the best negative predictor for survival [91].

In the same year, Palladini et al. examined the ability of NT-proBNP to predict an adverse outcome and to monitor the response to therapy. It emerged that NT-proBNP was significantly higher in patients with cardiac amyloidosis than in those without heart involvement and that a cut-off of 152 pmol/L had a sensitivity of 93% and a specificity of 90% for the diagnosis of heart infiltration. Patients with values above this cut-off were at least six times more likely to die than those with lower values. Moreover, NT-proBNP showed to be more accurate in reflecting therapy response, intended as amyloidogenic protein reduction, than echocardiographic parameters [92].

In 2004, Dispenzieri et al. retrospectively assayed NT-proBNP in sera from the same patients who had been included in their previous analysis [91], and designed a prognostic model which classified patients in three groups according to their positivity to none (stage I), one (stage II) or both (stage III) the markers, NT-proBNP and troponin T. Median survival decreased significantly with stage progression [93]. Subsequently, the same authors have retrospectively tested their scoring system in patients undergoing peripheral blood stem cell trans-plantation in order to verify the ability of the same model to identify patients with a more favorable prognosis and who would benefit more from the graft: patients at stage III had, also in this case, a worse prognosis than those at stage I or II [94].

Several subsequent studies have also confirmed the predictive ability of cardiac biomarkers for long-term survival [95,96]. Research has also focused on identifying which had the best diagnostic profile between natriuretic peptides and troponin. Palladini et al. in 2010 compared high-sensitivity cTnT (hs-TnT), NT-proBNP, and troponin I. All of the three markers were good survival predictors, but hs-TnT showed the highest accuracy. On the contrary, NT-proBNP proved to be the best marker to evaluate the response to treatment [97]. Apridonidze et al. found that at univariate analyses predictors of all-cause mortality included, among others, increased troponin and BNP, but, from Cox’s multivariate survival analysis, only troponin was a predictor of all-cause mortality [98].

Since long-term survival in patients with AL amyloidosis is based not only on the degree of cardiac involvement, but also on some characteristics of the plasma cell clone, Dispenzieri et al. in 2012 updated their prognostic staging system by incorporating into it a third marker: the difference between involved and uninvolved light chain. They obtained a new four-stage risk stratification system capable of predicting the survival of patients with AL amyloidosis at 5 years [99]. However, since high sensitivity assays for troponin T had not been used yet in their model, the same authors investigated whether replacing TnT with hs-TnT would improve its prognostic accuracy. It emerged that hs-TnT improved the prognostic value of the model and, moreover, that even when considered alone, hs-TnT could provide excellent information on prognosis. [100].

In conclusion, hs-TnT is currently used as a reference marker for cardiac AL amyloidosis. Values greater than 50-54 ng/L predict increased mortality rates and are strongly correlated with NYHA functional class, LVEF, and left ventricular wall thickness. As for NT-proBNP, it also remains an excellent prognostic indicator [7].

## 5. Conclusions

Cardiac biomarkers, natriuretic peptides and troponins in particular, have been investigated for their potential role as prognostic, predictive, diagnostic and monitoring clinical tools in multiple cardioncological fields.

They have proved to be useful and inexpensive in several clinical settings, however there are several gaps in the evidence which often do not yet allow to establish their precise role even in areas where they have been extensively investigated.

Regarding anthracycline-related cardiotoxicity it seems indisputable that troponin increases gradually during chemotherapy, at least in a relevant percentage of the patients treated. However, it is not clear why troponin does not increase in all of the patients, even when they have similar underlying conditions at baseline. Troponin was proven to have only a high negative predictive value towards clinically detectable cardiotoxicity, but not a high positive predictive value, as emerged from the only meta-analysis carried out so far; this is also due to the use of different cut-offs in the various clinical trials, since a specific one for cardiotoxicity has not been identified yet, but there are probably other unknown patients-related factors that contribute to the onset of LVD only in a minority of those who experience a troponin release during chemotherapy. Therefore, a troponin rise during anthracycline courses remains still difficult to precisely interpret. Nevertheless, there is evidence that the increase in troponin itself, which certainly represent the onset of subclinical myocardial damage due to cardiotoxicity, may be sufficient to justify the preventive use of cardioprotective therapies.

As for Trastuzumab related cardiotoxicity, the evidence shows that this drug does not induce a release of troponin; rather, some patients would experience an increase in this marker during the very first cycles only as a consequence of deferred toxicity from previous anthracycline therapy. Therefore, troponin would not represent a useful indicator of the direct cardiotoxic effects of Trastuzumab.

Troponin was proven to be a sensitive marker for ICIs related myocarditis, but not very specific, with the consequent risk that an isolated increase of the marker could lead clinicians to improperly interrupt chemotherapy; for this reason, and also in view of the rather low incidence of this adverse event, the routine use of troponin for screening purposes in this setting is still a matter of debate and is not currently indicated by guidelines.

As far as radiotherapy is concerned, any troponin increase recorded during treatment has not been clearly correlated with long-term adverse outcomes, given the long latency between the therapy itself and the onset of related cardiovascular diseases.

Regarding the ability to predict POAF in patients undergoing lung cancer surgery, NT-proBNP was proven to have good sensitivity and specificity, while results for troponin are in contrast.

Elevated baseline NT-proBNP and hsTn appear to be related to worse outcomes in patients undergoing androgen deprivation for prostate cancer, particularly when GnRH agonists are used; however higher quality evidence is needed.

With regard to the ability of cardiac biomarkers to predict, diagnose and/or monitor the course of cancer-related cardiovascular diseases not on iatrogenic bases, their usefulness appears greater and their role more defined than those demonstrated for the prediction of cardiac damage induced by oncological treatments.

NT-proBNP was proven to be extremely sensitive and specific for the detection of severe heart valves dysfunction related to syndromic carcinoid, so much so that a precise cut-off (260 pg/mL) has been identified to select patients deserving a more in-depth study with cardiovascular imaging.

Finally, concerning AL amyloidosis, both NT-proBNP and hsTnT were proven to be effective for diagnostic purposes and in predicting outcome, so much so that they have been incorporated into various prognostic models.

## 6. Future Perspectives

Given what has been mentioned so far, several objectives for the future emerge, among which are the following:

- trying to individuate a troponin cut-off that is specific for anthracycline cardiotoxicity, which should have at least a high negative predictive value for future LVD development in order to identify patients who can benefit from a early preventive cardioprotective therapy; in particular, research should focus on that subset of patients with multiple baseline cardiovascular risk factors or who receive high cumulative doses of anthracyclines or who are planned to undergo multiple cardiotoxic treatments in succession (i.e., anthracyclines followed by trastuzumab, anthracyclines followed by mediastinal radiotherapy, etc.), since those are the patients more at risk of developing LVD;
- clearly establishing whether a screening strategy based on serial troponin dosage for ICIs myocarditis is actually useful, taking into account the low incidence of the phenomenon and the frequent cases of isolated aspecific hyper-troponinemia, or if instead it induces only an increase in inappropriate therapy discontinuation rate;
- establishing whether troponin increase during treatment with thoracic radiotherapy correlates with the future development of post-actinic heart disease, although this objective is difficult to achieve given the long interval between therapy end and the eventual development of the disease;
- more quality evidence is needed to establish the utility of NT-proBNP in predicting which patients undergoing androgen deprivation are at risk of developing cardiovascular complications and therefore deserve closer cardiological follow-up and the implementation of preventive cardiac therapy.

## Figures and Tables

**Table 1:** Causes of troponin elevation. Modified from Thygesen et al. [11].

Main Phatophysiological Mechanism	Pathology
Injury related to primary myocardial ischemia	Plaque rupture
	Intraluminal coronary artery thrombus formation
Injury related to supply/demand imbalance of myocardial ischemia	Tachy-/brady-arrhythmias
	Aortic dissection or severe aortic valve disease
	Hypertrophic cardiomyopathy
	Cardiogenic, hypovolemic, or septic shock
	Severe respiratory failure
	Severe anemia
	Hypertension with or without LVH
	Coronary spasm
	Coronary embolism or vasculitis
	Coronary endothelial dysfunction without significant CAD
Injury not related to myocardial ischemia	Cardiac contusion, surgery, ablation, pacing, or defibrillator shocks
	Rhabdomyolysis with cardiac involvement
	Myocarditis
	Cardiotoxic agents, e.g., anthracyclines, herceptin
Multifactorial or indeterminate myocardial injury	Heart failure
	Stress (Takotsubo) cardiomyopathy
	Severe pulmonary embolism or pulmonary hypertension
	Sepsis and critically ill patients
	Renal failure
	Severe acute neurological diseases, e.g., stroke, subarachnoid
	hemorrhage
	Infiltrative diseases, e.g., amyloidosis, sarcoidosis
	Strenuous exercise
